# Urban health inequities and healthy longevity: traditional and emerging risk factors across the cities and policy implications

**DOI:** 10.1007/s40520-025-03052-1

**Published:** 2025-05-07

**Authors:** Stefano Cacciatore, Sofia Mao, Mayra Villalba Nuñez, Claudia Massaro, Luigi Spadafora, Marco Bernardi, Francesco Perone, Pierre Sabouret, Giuseppe Biondi-Zoccai, Maciej Banach, Riccardo Calvani, Matteo Tosato, Emanuele Marzetti, Francesco Landi

**Affiliations:** 1 Department of Geriatrics, Orthopedics and Rheumatology, Università Cattolica del Sacro Cuore, Largo F. Vito 1, 00168 Rome, Italy; 2 Fondazione Policlinico Universitario “Agostino Gemelli” IRCCS, Largo A. Gemelli 8, 00168 Rome, Italy; 3 Department of Life Sciences and Public Health, Università Cattolica del Sacro Cuore, Largo F. Vito 1, 00168 Rome, Italy; 4 Fundación Azikna, Fraga 432, C1427, Ciudad Autónoma de Buenos Aires, Argentina; 5 Consejo de Cardiogeriatría, Sociedad Argentina de Cardiología, Azcuénaga 980, C1115 Ciudad Autónoma de Buenos Aires, Argentina; 6 Department of Medical-Surgical Sciences and Biotechnologies, Sapienza University of Rome, Corso Della Repubblica 79, 04100 Latina, Italy; 7 UOC UTIC Emodinamica e Cardiologia, Ospedale Santa Maria Goretti, Via Lucia Scaravelli, 04100 Latina, Italy; 8 Cardiac Rehabilitation Unit, Rehabilitation Clinic “Villa Delle Magnolie”, Via Ciummiento, 37, 81020 Castel Morrone, Caserta Italy; 9 Sorbonne University, ACTION Study Group, Inserm UMRS1166, Heart Institute, Pitié-Salpetriere Hospital, 47–83 Bd de L’Hôpital, 75013 Paris, France; 10 Maria Cecilia Hospital, GVM Care & Research, Via Corriera, 1, 48033 Cotignola, Italy; 11 Faculty of Medicine, The John Paul II Catholic University of Lublin, Aleje Racławickie 14, 20-950 Lublin, Poland; 12 Department of Preventive Cardiology and Lipidology, Medical University of Lodz (MUL), Rzgowska 281/289, 93-338 Lodz, Poland; 13 Ciccarone Center for the Prevention of Cardiovascular Disease, Division of Cardiology, Department of Medicine, Johns Hopkins University School of Medicine, 600 N. Wolfe St, Baltimore, MD 21287 USA

**Keywords:** Healthy aging, Cardiovascular risk factors, Urbanization, Environment, Sustainable development, Climate change, Pollution, Mental health, Healthcare access, Health promotion strategies

## Abstract

Urbanization is reshaping global health, with over 55% of the world’s population residing in urban areas, a figure projected to reach 68% by 2050. This demographic shift presents significant challenges and opportunities for public health, as urban environments exacerbate health disparities rooted in social determinants of health, such as economic stability, education, neighborhood conditions, and access to healthcare. Rapid urban growth, particularly in low- and middle-income countries, has led to the emergence of inequitable living conditions, environmental hazards, and limited access to essential health services, contributing to the early onset of multimorbidity and rising non-communicable disease burdens. Urbanization-driven factors such as obesogenic environments, sedentary lifestyles, air pollution, and inadequate sleep exacerbate cardiovascular and metabolic risks, while social exclusion, overcrowding, and inadequate mental health services heighten vulnerabilities. Emerging risks, including urban heat islands, noise pollution, and exposure to endocrine-disrupting chemicals, further compound urban health inequities. Effective mitigation requires multi-sectoral policies that prioritize health-promoting infrastructure, reduce environmental pollutants, foster equitable healthcare access, and address systemic barriers affecting marginalized groups. This review explores the intersections between urbanization and health inequities, emphasizing the importance of addressing traditional and emerging risk factors across the lifespan. Policy implications include promoting green infrastructure, enhancing urban mobility, expanding mental health care, and leveraging participatory governance to foster resilient and inclusive cities. By adopting an integrated approach that prioritizes social equity and sustainability, cities can mitigate health disparities and create healthier, more inclusive urban environments that support the well-being of all residents.

## Introduction

**Fig. 1:**
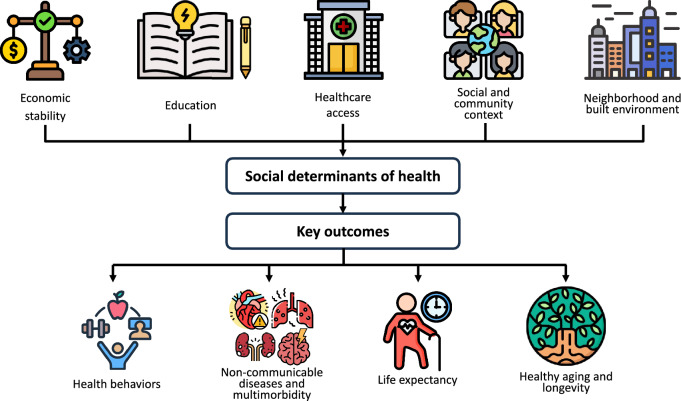
The social determinants of health and their influence on key health outcomes

Urbanization, the process by which populations increasingly concentrate in cities, is transforming the global landscape at an unprecedented pace. Over 55% of the world's population currently resides in urban areas, a figure projected to rise to 68% by 2050 [1]. This demographic shift represents not only a hallmark of modern development but also a critical challenge and opportunity for global health. As cities grow, particularly in developing regions, guiding urban development to support health and well-being has never been more urgent. Yet, for many of the 4.2 billion people living in urban areas, the reality remains starkly different [2]. Urban health dynamics are shaped by a complex interplay of individual, social, and environmental factors. Socioeconomic disparities lie at the heart of urban health inequities, influencing access to education, quality employment, healthcare, and healthy living conditions. The socioeconomic stratification typical of urban settings often dictates health outcomes, with disadvantaged groups facing an earlier onset of chronic diseases and higher rates of multimorbidity [3]. Social determinants of health (SDOH), the conditions in which people are born, grow, live, work, and age, play a pivotal role in shaping these outcomes (Fig. 1). Key domains of SDOH include economic stability, neighborhood and built environment, education, social and community context, and access to healthcare. The built environment, encompassing human-made surroundings such as neighborhoods, parks, transportation systems, and waste management infrastructure, significantly influences health outcomes [4]. Poorly designed urban spaces often exacerbate health risks, limiting opportunities for active living, and contributing to air, noise, water, and soil pollution [1, 2]. These environmental factors disproportionately impact marginalized populations, exposing them to poor living conditions and increased the risk of disease and disability throughout their lives [5].

This review examines the intersection of urbanization, health inequities, and disease patterns, highlighting both traditional and emerging risk factors. Eventually, it aims to identify strategies for fostering healthier, more equitable urban environments that support well-being across the lifespan.

## Traditional risk factors and urban health inequities

**Fig. 2:**
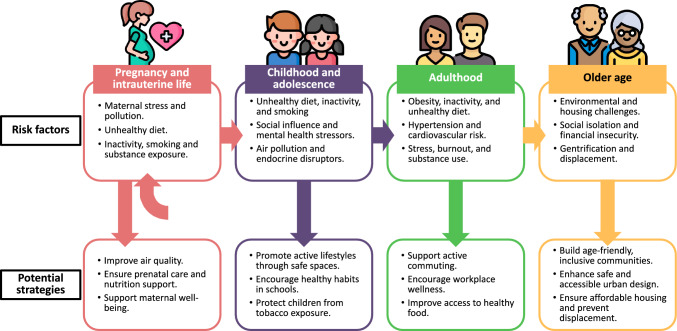
Urbanization-linked risk factors for non-communicable disease risk progression across the lifespan and potential strategies for prevention

Global population aging is a significant trend, with people living longer due to advancements in healthcare, improved socioeconomic conditions, and better access to medical technologies [6]. Life expectancy is rising not only in high-income countries but also in low- and middle-income countries (LMICs), driven by public health improvements, vaccination programs, and reductions in infectious diseases [7]. However, this increase in lifespan is often not matched by a corresponding increase in healthspan, that is the years of life spent in good health, free from chronic illness. In epidemiology it is called healthy life years [8, 9]. The average gap between healthy life years and life expectancy is estimated to be 9.6 years, ranging from 6.55 years in Lesotho to 12.4 years in the USA [8]. Multimorbidity, defined as the presence of two or more chronic conditions, affects 37.2% of individuals worldwide and represents a significant challenge to healthcare systems worldwide [10]. Although multimorbidity is commonly associated with aging, it also affects younger populations, particularly in LMICs, where social determinants play a key role in health disparities. Studies indicate that individuals with fewer socioeconomic resources experience the onset of multimorbidity 10–15 years earlier than their wealthier counterparts [3]. These trends highlight the need for lifelong strategies to promote healthy aging and reduce chronic disease risks (Fig. 2).

Primordial prevention, a concept introduced by Strasser in 1978, emphasizes addressing the root causes of disease by preventing the emergence of harmful environmental, social, and behavioral conditions [11]. In line with this approach, in 2010 the American Heart Association (AHA) introduced Life’s Simple 7 (LS7), a framework of seven key metrics to promote cardiovascular health through healthy lifestyle choices, such as regular physical activity, a healthy diet, smoking cessation, and effective management of body weight, cholesterol, blood pressure, and blood glucose levels [12]. In 2022, the AHA expanded this framework with Life’s Essential 8 (LE8), adding new components like sleep health and refining existing metrics LE8 also emphasized the role of social, ecological, and psychological determinants of health, reinforcing the importance of a holistic"mind–body-heart"connection and advocating for cardiovascular health promotion at every stage of life [13]. While primarily focused on cardiometabolic health, LE8 addresses risk factors relevant to other noncommunicable diseases (NCDs), including dementia, and multimorbidity [14–16]. There are also other attempts to further extend this important issue, including the recent International Lipid Expert Panel (ILEP) Simple Tips for the healthy heart that additionally put the emphasis on the alcohol consumption (there is no healthy amount of alcohol), stress (especially chronic stress), nonadherence to medical prescriptions and lack of health education, and lipoprotein(a) as independent cardiovascular risk factors [17].

However, applying frameworks like LE8 or the ILEP Simple Tips in urban environments reveals persistent health inequities. Longitudinal studies, such as Whitehall II and UK Biobank, have shown that approximately one-third of the increased risk of dementia among socioeconomically disadvantaged groups can be attributed to differences in cardiovascular health. The relationship between cardiovascular health and social disparities is even more pronounced for stroke and coronary heart disease, aligning with prior research [18]. This evidence underscores the importance of addressing traditional risk factors, particularly among socioeconomically disadvantaged groups, to reduce health disparities and decrease the burden of chronic diseases in cities.

### Health behaviors within the cities: healthy eating, physical activity, nicotine exposure, sleep health

LE8 define health behaviors as lifestyle choices or actions that individuals can actively adopt or change to improve their cardiovascular health. They are focused on modifiable behaviors that can lead to better outcomes and include healthy eating, physical activity, nicotine exposure, sleep health [13]. Urbanization significantly influences health behaviors, often amplifying disparities among different socioeconomic groups.

Urban environments shape dietary habits through food availability, cultural preferences, and economic factors. Higher-income urban residents typically have access to a wide variety of nutritious foods, while lower-income populations face significant barriers to healthy eating due to"food deserts", areas where residents have limited or no access to affordable, nutritious options such as fresh fruits, vegetables, and whole foods. These areas often overlap with "food swamps", where convenience stores and fast-food outlets dominate, offering mostly processed, high-fat, high-sugar foods. The absence of supermarkets or grocery stores in these neighborhoods profoundly impacts the eating habits and overall health of lower-income communities [19]. Another factor shaping eating patterns in urban areas is the increasing "westernization" of diets, which is considered a major contributor to the rising prevalence of NCDs. Long-term follow-up studies have shown a significant association between urbanization and income levels with a decline in the consumption of traditional foods and a shift toward increased intake of highly processed foods, refined sugars, unhealthy fats, and red or processed meats [20, 21]. The promotion of restrictive diets, such as ketogenic and carnivore diets, through social media platforms has significantly influenced the public's approach to weight management and cardiovascular health [22]. These diets, which are characterized by high-fat and low-carbohydrate intake, have shown short-term benefits for weight loss and glycemic control in some studies, but concerns remain regarding their potential long-term cardiovascular risks. For instance, some studies highlight the benefits of ketogenic diets in improving lipid profiles, though there is evidence of increased low-density lipoprotein (LDL) levels and potential associations with dyslipidemia and cardiac fibrosis [23]. The growing trend of using semaglutide, a glucagon-like peptide 1 receptor agonist, for rapid weight loss without medical supervision raises concerns regarding adverse outcomes. While semaglutide has been shown to reduce cardiovascular events in populations with obesity, its use outside medical guidance can lead to significant risks, including gastrointestinal side effects, gallbladder issues, and unintended muscle loss [24]. Additionally, misuse without accompanying lifestyle changes, including implementation of physical exercise, may exacerbate muscle wasting and impair body composition [25]. Reports of drug misuse highlight the need for careful monitoring, as semaglutide has been associated with a higher incidence of adverse effects when taken outside prescribed regimens [26]. Additionally, a retrospective cohort in Israel revealed that individuals from higher socioeconomic backgrounds had shorter times to sodium-glucose co-transporter 2 inhibitors prescriptions, suggesting that economic wealth can influence access, including for appropriate prescription, and potential overuse [27].

The built environment plays a critical role also in determining physical activity levels. A large study involving 9472 American adolescents and young adults by Armstrong et al. [28] identified significant differences in physical activity levels across income and ethnic groups. Wealthier neighborhoods often feature parks, green spaces, and pedestrian-friendly infrastructure, encouraging active lifestyles. In contrast, low-income areas frequently lack safe, accessible recreational spaces, limiting opportunities for physical activity [29]. Urban sprawl, which prioritizes vehicular traffic over walkability, further restricts active living, especially in marginalized communities. Evidence shows that compact, walkable neighborhoods with diverse land use and efficient public transport foster higher physical activity levels, while concerns about safety, inadequate lighting, and poor infrastructure deter outdoor exercise in disadvantaged areas [30, 31]. Income inequality, reflected in economic disparities that limit access to fitness centers, organized sports, and transportation to areas with better facilities, plays a pivotal role in shaping physical activity levels.

Urbanization influences also smoking behaviors through increased tobacco accessibility and targeted marketing in low-income neighborhoods. Higher densities of tobacco retail outlets in disadvantaged areas contribute to elevated smoking rates, particularly among marginalized populations [32, 33]. SDOH, such as unemployment, community safety and exposure to violence, lower social cohesion and limited recreational spaces, further drive tobacco use as a coping mechanism for economic and emotional stress [34, 35]. In densely populated urban settings, exposure to secondhand smoke in multi-unit housing compounds health risks, including respiratory illnesses [36]. Electronic Nicotine Delivery Systems (ENDS), including exposure to secondhand aerosol, present significant health risks and have been associated with respiratory and cardiovascular diseases (CVDs) [37, 38]. Research indicates that accessibility to vape shops, peer pressure, and targeted advertising in urban settings contribute to increased ENDS use among adolescents and adults. High-income urban areas, in particular, report higher experimentation rates, likely due to increased disposable income and more frequent exposure to promotional materials. Additionally, studies reveal that the proximity of ENDS retailers to schools and public spaces correlates with higher usage rates among youth [39].

Sleep health is a crucial yet frequently overlooked determinant of overall well-being [13]. In urban settings, disparities in sleep quality and duration are strongly influenced by socioeconomic conditions and neighborhood environments and disproportionately impact marginalized communities [40]. Low-income residents frequently experience disrupted sleep due to overcrowded housing, crime, noise pollution, and inadequate infrastructure. In contrast, wealthier neighborhoods typically offer quieter environments, green spaces, and safer streets, which support healthier sleep patterns [41]. Occupational demands, such as shift work, more common in low-income populations, exacerbate sleep deprivation and circadian misalignment [42]. Limited access to healthcare services for identifying lifestyle, psychological, and organic sleep disturbances, along with inadequate sleep education, further exacerbates these issues [43].

Alcohol consumption remains a significant public health issue in urban areas, with concerning trends in both quantity and patterns of use. There is no safe amount of alcohol, as even moderate consumption is associated with increased risks of chronic diseases, such as liver damage and CVDs, as well as heightened vulnerability to mental health disorders [44]. Despite global efforts to raise awareness, alcohol consumption rates have not declined in recent decades [17, 45]. Instead, there has been a shift in the types of alcohol consumed, rather than a reduction in overall intake. In many urban centers, a common trend known as "weekend alcoholism" has emerged, particularly among individuals employed in large industries, where heavy episodic drinking is normalized as a form of stress relief [44, 46]. Alcohol consumption is heavily influenced by social determinants such as education, occupation, income, housing stability, social marginalization, cultural norms and access to healthcare, all of which shape patterns of use and associated health outcomes.

### Health factors within the cities: overweight and obesity, blood glucose, blood lipids and blood pressure

Health factors, defined as biological or physiological markers that indicate the body’s current cardiovascular health status, are measurable variables often influenced by health behaviors. According to LE8, these factors include body weight (overweight and obesity), blood glucose levels, blood lipid levels, and blood pressure. Inequities in access to healthy, nutritious food, combined with insufficient physical activity, play a critical role in driving these abnormalities. Together, these factors contribute to the development of metabolic syndrome, a condition that significantly increases the risk of heart disease, stroke, and diabetes. According to a study conducted in India, the prevalence of metabolic syndrome is significantly higher in urban populations (54.8%) compared to rural populations (46.2%), highlighting the impact of urbanization on metabolic health disparities [47].

Research on the prevalence of obesity in rural and urban settings, however, often yields inconsistent results due to varying determinants across different demographic groups. For example, children and adolescents in rural areas face up to 30% greater odds of being overweight or obese compared to their urban counterparts [48]. In contrast, urban populations may experience higher obesity rates among low-income women and minority groups due to socioeconomic constraints, such as food insecurity, lack of green spaces, and sedentary lifestyles [49, 50]. These disparities highlight the need for context-specific approaches, as determinants of obesity, such as income, gender, age, and cultural norms, differ widely across population groups, influencing trends in both rural and urban areas.

The prevalence and outcomes of diabetes, dyslipidemia, and hypertension in the cities are closely linked to disparities in healthcare access and education. Low-income individuals often lack insurance or face prohibitive costs for medications and diagnostic tools, resulting in inconsistent monitoring and poor treatment adherence [51–53]. On the other hand, health literacy disparities also hinder effective management, as individuals with limited education may struggle to understand the importance of consistent monitoring and lifestyle changes [53, 54]. A study conducted in Mexico City found that diabetes knowledge and access to self-care tools were directly associated with socioeconomic status, with poorer individuals experiencing significantly worse glycemic outcomes [55]. Additionally, chronic stress, poor sleep, and irregular work hours, common challenges in low-income urban populations, further disrupt glucose and blood pressure regulation [56]. Psychosocial stress driven by factors such as workplace demands, socioeconomic pressures, and cultural changes is strongly associated with an increased risk of developing hypertension. A meta-analysis revealed that individuals experiencing chronic psychosocial stress had 2.4 times higher odds of developing hypertension [57]. Women, older adults, and marginalized groups face greater risks due to caregiving responsibilities, limited healthcare access, and systemic inequities [58]. Regarding blood pressure control, among dietary determinants, inexpensive, processed foods high in sodium and low in potassium contribute to the growing burden of hypertension in underserved communities. A meta-analysis involving 134,916 participants from low- and middle-income countries found that excessive salt consumption increased the likelihood of hypertension by 42% in urban areas and by 7% in rural areas for each additional gram of salt consumed. The prevalence of high salt intake, however, ranged from 21.3 to 89.5% in both urban and rural settings, underscoring the widespread nature of this dietary risk factor. [59]. Regarding blood lipid control, research shows that the prevalence of dyslipidemia in urban areas is often higher compared to rural regions, but the specific lipid profile abnormalities can vary across settings. A study in China found a similar overall prevalence of dyslipidemia in rural and urban areas (43.2% vs. 43.3%) [60]. In contrast, data from Pakistan highlighted significantly higher urban prevalence rates for hypercholesterolemia (39.3%), hypertriglyceridemia (48.9%), and low HDL-C (87.4%) [61]. Similarly, findings from the CRONICAS Cohort Study in Peru reported a higher prevalence of low high-density lipoprotein in urban regions and elevated LDL in semi-urban areas [62]. Socioeconomic factors influence not only lipid profiles but also the progression of atherosclerotic disease and mortality. A population-based cohort study of 11,946 dyslipidemia patients in Korea revealed that residents of less affluent neighborhoods faced a 64% higher mortality risk compared to a 48% increase in more affluent areas [63]. Similarly, a study from Poland demonstrated that urbanization-related factors, such as housing density and commercial developments, significantly impacted coronary artery disease progression in patients with dyslipidemia [64]. Taking into account the available data, elevated lipoprotein(a) is an independent cardiovascular risk factor with a prevalence ranging from 15 to 25%. This makes it the fourth most prevalent cardiovascular risk factor, following lipid disorders, hypertension, and smoking. However, it remains largely unrecognized, particularly in rural areas, where its underdiagnosis is especially pronounced [65, 66].

## Emerging risk factors and urban health inequities

While cities often serve as hubs of economic opportunity and provide advanced healthcare services, there are also emerging challenges that deepen health disparities and escalate the prevalence of chronic conditions (Table 1).

**Table 1:** Summary of environmental and social factors influencing urban health inequities, their impacts, illustrative examples, and potential remedial actions

Factor	Impact	Potential remedial actions
Air pollution	Increases respiratory and cardiovascular diseases	Stricter emission controls Promotion of public transportation Increase green spaces and reduce pollution
Exposure to environmental hazards	Increases the risk of chronic diseases, including cancer	Stricter environmental regulations Regular monitoring Resident relocation from high-risk areas
Inadequate housing	Contributes to respiratory issues and mental health problems	Ensure proper housing and ventilation Reduce overcrowding
Lack of green spaces	Reduces opportunities for physical activity and mental relaxation	Foster urban parks and community gardens Integrate green roofs and walls in buildings
Limited access to healthcare services	Delays in treatment and preventive care, worsening health outcomes	Establish community health centers Promote mobile clinics Improve access to healthcare facilities
Noise pollution	Causes stress, sleep disturbances and cardiovascular issues	Enforce noise regulations Design urban layouts to minimize noise exposure Promote the use of noise barriers
Poor sanitation	Leads to the spread of infectious diseases	Invest in sewage systems Regular waste collection Public health education campaigns
Social isolation	Affects mental health, leading to depression and anxiety	Create community centers Promote social activities Design public spaces for interaction
Traffic congestion	Increases air pollution and stress, leading to respiratory and mental health issues	Develop public transportation Promote cycling and walking Apply traffic management strategies
Urban heat islands	Elevates temperatures, increasing heat-related illnesses	Increase urban greenery Use reflective building materials Design for natural ventilation

### Environmental risk factors

The concept of the exposome encompasses the totality of environmental exposures an individual encounters throughout their lifetime, including physical, chemical, and biological factors that influence health. Among these, air pollution, noise pollution, and other environmental risk factors are key components of the exposome that significantly contribute to the overall burden of disease [67]. Urban areas often face significant environmental health challenges, with air pollution being a primary concern. Exposure to air pollution, particularly fine particulate matter (PM2.5), nitrogen dioxide, and ozone, is strongly associated with an increased risk of both cardiovascular and pulmonary diseases, contributing to higher mortality rates. PM2.5 can penetrate deep into the alveoli, enter the bloodstream, and trigger systemic inflammation and oxidative stress, leading to endothelial dysfunction, a key event in atherogenesis, as well as persistent airway inflammation, alveolar damage, and the progression of chronic obstructive pulmonary disease and asthma. Nitrogen dioxide, primarily emitted from vehicular traffic, enhances vascular oxidative damage, impairs nitric oxide bioavailability, and induces vasoconstriction and hypertension, while also impairing lung function and increasing airway hyperreactivity and the risk of respiratory infections. Ozone exposure promotes the generation of reactive oxygen species, which contribute to inflammatory responses, autonomic nervous system imbalance, and arrhythmias, while also exacerbating asthma by inducing bronchoconstriction, airway inflammation, and epithelial damage. Collectively, these pollutants drive the progression of atherosclerosis, increase blood coagulability, worsen respiratory symptoms, and heighten susceptibility to acute events such as myocardial infarction, stroke, and respiratory exacerbations, emphasizing the critical importance of air quality improvement in preserving both cardiovascular and respiratory health [68, 69]. Chronic exposure to pollutants has also been associated with neurodegenerative diseases such as Alzheimer’s and Parkinson’s diseases, pregnancy complications, and reduced birth weights [70–72]. Low-income urban neighborhoods, often near industrial zones and highways, bear the brunt of poor air quality [73]. Compounding these risks is indoor air pollution, which arises from everyday sources such as cooking fuels, incense, and household cleaning products. In many regions, particularly where wood-burning stoves or open fires are commonly used for cooking and heating, indoor pollutants can reach dangerous levels, further exacerbating respiratory and cardiovascular health risks [74]. Seasonal variations and climate change have amplified the impacts of urban air pollution, intensifying its harmful effects on human health. During certain seasons, temperature fluctuations, humidity levels, and atmospheric pressure changes can increase pollutant concentrations, creating prolonged exposure periods, particularly in densely populated cities. Additionally, climate-related phenomena, such as heatwaves and wildfires, contribute to higher levels of particulate matter and ground-level ozone [75]. Urban heat islands (UHIs), driven by heat-retaining materials such as asphalt and concrete, amplify health risks during heatwaves, contributing to heatstroke and cardiovascular strain. Vulnerable groups, including older adults, children, and outdoor workers, are particularly at risk [76]. The effects of UHIs are further intensified by rising global temperatures, which have been associated with increased hospitalizations and higher mortality rates during extreme heat events [77]. In addition to physical health impacts, the UHI effect worsens mental and overall health issues by disrupting sleep patterns and elevating stress levels. Sustained exposure to elevated nighttime temperatures has been linked to impaired cognitive performance and increased anxiety, especially among urban youth and low-income populations [78].

Noise pollution from traffic, construction, and industrial activity is a growing concern in urban areas, contributing not only to hypertension and CVDs but also to increased levels of anxiety, sleep disturbances, and cognitive decline. Chronic exposure to high noise levels has been linked to elevated cortisol levels, triggering a sustained stress response that further exacerbates these health risks [79, 80]. Similarly, poor water quality, often caused by aging infrastructure, industrial discharge, and inadequate water treatment, significantly raises the risk of gastrointestinal diseases and waterborne infections. This issue is particularly severe in informal settlements, where limited access to clean water and safe sanitation leads to widespread contamination of food and drinking water and incidence of water-borne diseases [81].

### Urbanization-linked epidemiological shifts and technological and digital risk factors

Urbanization has shifted health behaviors, especially among youth, who face rising risks from sedentary lifestyles, poor diets, and increased screen time [82]. Urban design that prioritizes cars over walkable spaces, reducing opportunities for physical activity, contribute to the growing prevalence of sedentary behavior. The availability and marketing of processed, calorie-dense foods often displace healthier, nutrient-rich options, particularly in lower-income neighborhoods. Increased screen time exacerbates these challenges by encouraging prolonged inactivity and exposure to advertising for unhealthy food and beverages, further influencing dietary choices. Excessive digital device use not only disrupts natural sleep patterns through prolonged exposure to blue light but also shortens sleep duration, a factor closely linked to obesity and other metabolic disorders. This disruption in circadian rhythms, coupled with insufficient physical activity, creates a fertile ground for early-onset conditions like type 2 diabetes and hypertension [83]. Mental health challenges are also prevalent, with academic pressures and urban stressors fueling higher rates of anxiety and depression [84].

Endocrine-disrupting chemicals (EDCs) in urban environments, found in plastics, cosmetics, and industrial waste, interfere with hormonal regulation and contribute to obesity, diabetes, and reproductive health disorders [85, 86]. Adolescents are particularly vulnerable, as EDC exposure during growth stages can cause long-term health disruptions [87]. Inadequate disposal of electronic waste in low-income areas increases exposure to heavy metals such as lead and mercury, further increasing health risks [88].

### Infectious disease epidemics and extreme weather

Urban centers, interconnected by global trade and travel, have become hotspots for infectious disease transmission. The COVID-19 pandemic illustrated how densely populated cities act as hubs for rapid spread, particularly in settings with inadequate health infrastructure. A study by Aboukorin et al. [89] on European cities across England, Germany, and Italy sheds light on how urban planning characteristics exacerbate or mitigate disease transmission. The authors found that intra-city connectivity, particularly reliance on public transportation, significantly correlated with higher infection rates. Milan, Italy for instance, exhibited both a high public transit dependency and one of the highest infection rates, illustrating how interconnected transport networks can foster rapid contagion. In contrast, cities like Cottbus, Germany, which had lower public transport usage, recorded significantly fewer cases. The study also noted that factors such as population size and density played roles, though their impact was less pronounced than connectivity. Beyond structural factors, unsustainable practices, such as live animal markets, heighten the risk of zoonotic spillovers. These markets, where live animals are kept in close quarters and often in poor sanitary conditions, create an ideal environment for the transmission of pathogens between species [90].

Disparities in education within cities and between urban and rural areas significantly influenced public health outcomes during the pandemic. Limited education contributed to the spread of misinformation, heightened vaccine hesitancy, and reduced health literacy, which in turn led to lower vaccination rates and poorer adherence to public health measures. Communities with lower educational attainment, often marked by socioeconomic marginalization, experienced disproportionate impacts from COVID-19, including higher rates of infection, morbidity, mortality, and post-acute sequelae compared to wealthier, better-educated urban areas [91, 92].

Climate-related disasters, such as floods and heatwaves, place significant strain on urban health systems, overwhelming their capacity to respond effectively. Flooding can lead to outbreaks of waterborne diseases due to contaminated water supplies, while extreme heat exacerbates respiratory conditions and increases the risk of heat-related illnesses [93]. Together, these events create a cascade of public health challenges, disproportionately affecting vulnerable populations and highlighting the urgent need for resilient infrastructure and adaptive health strategies [94].

### Structural factors, population aging and globalization

Access to healthcare remains a significant challenge in urban slums and informal settlements, where internal and external migrants, along with marginalized individuals, often reside. Overcrowded clinics, financial constraints, and the absence of health insurance contribute to delays in both preventive care and necessary treatments [95]. Community-driven initiatives have shown promise in reducing urban health disparities by empowering residents, improving access to resources, and fostering collaboration. Approaches like community-based participatory research involve locals in identifying challenges and co-creating solutions, enhancing sustainability and trust. Examples include the Chicago Breast Cancer Task Force, which improved screening access and cut mortality disparities by 35%, and an East Los Angeles youth program that converted corner stores into healthy food outlets, framing nutrition as a social justice issue. Media interventions in Massachusetts also reshaped public health narratives, raising awareness of social determinants of health [96].

Population aging poses additional challenges for urban health systems as cities adapt to a growing number of older residents. This is especially true in emerging countries with growing populations, an expanding proportion of older adults, and rapidly increasing urbanization [97]. While urban areas offer advanced healthcare infrastructure, they can also amplify disparities in healthcare access, social inclusion, and environmental exposure. Older adults often face chronic conditions which require long-term, continuous care, and are particularly vulnerable to factors such as air pollution and heat stress, especially those living with physical or social frailty, or both [98]. Research from Bucharest underscores the importance of inclusive policies that enhance financial security, healthcare access, and public spaces to promote active aging [99]. Expanding green infrastructure has also proven effective in mitigating environmental risks, while creating spaces that encourage physical activity and social interaction [100]. Equity-focused interventions, such as integrating geriatric care into primary healthcare, offering affordable housing, and fostering community engagement, are essential. Lessons from rapidly aging societies like China highlight the importance of aligning social support systems with healthcare reforms to support healthy aging [101]. Moreover, digital inclusion is vital for ensuring that older adults can participate in health monitoring and benefit from technological support services [102].

Rapid urban migration places additional strain on housing, healthcare, and sanitation systems, especially in low- and middle-income countries (Table 2). Both internal and external migrants often settle in densely populated areas with inadequate infrastructure, increasing their vulnerability to infectious diseases like tuberculosis and diarrheal illnesses. Overcrowding also facilitates the spread of vector-borne diseases and heightens mental health risks due to increased stress and diminished privacy [103]. In Delhi, India, studies have shown that health-seeking migrants frequently experience delays in accessing care due to unfamiliarity with healthcare services and discrimination [104]. Late-life migration, often for family reunification, presents unique challenges. Many aging migrants who join relatives in host countries struggle to adapt to new cultural contexts and healthcare systems. Studies of older Chinese immigrants in Australia and Canada reveal that while family networks play a critical role in their integration, they often face difficulties in maintaining autonomy and navigating unfamiliar medical services [105]. For many older migrants, the aging process is compounded by the challenges of migration, resulting in a"double burden"that accelerates health decline [106]. Language barriers are a significant concern, affecting their ability to describe symptoms, follow medical instructions, and access timely care [107]. Research on Turkish migrants in the Netherlands illustrates how limited language proficiency fosters dependency on family members for translation, reducing independence and privacy [108]. This issue is particularly problematic in diagnosing and managing conditions such as dementia. Qualitative data from the ImmiDem project in Italy highlights the barriers that older migrants face, including language difficulties, cultural misunderstandings, and limited access to healthcare services. These factors contribute to diagnostic delays, often preventing timely interventions that could slow the progression of cognitive decline [109]. Traditional cognitive assessments like the Mini-Mental State Examination may not account for cultural and educational differences, whereas culturally inclusive tools like the Rowland Universal Dementia Assessment Scale offer more accurate evaluations but remain underutilized [110]. Culturally sensitive care is crucial for managing dementia and other complex conditions among migrant populations [111]. For example, a study on Moroccan migrants with dementia in Belgium revealed significant challenges, such as a lack of culturally competent healthcare providers and a heavy reliance on informal family support networks [112]. Addressing these gaps requires healthcare systems to consider linguistic, cultural, and religious factors to provide holistic and inclusive care for aging migrant communities.

**Table 2:** Migration and urban health challenges

Aspect	Challenges	Implications for health	Potential strategies
Access to healthcare	Language barriers Lack of documentation Fear of discrimination	Delayed care Untreated chronic conditions Poor preventive care	Multilingual care and mediators Inclusive policies for undocumented migrants
Housing and living conditions	Overcrowding Poor sanitation Informal housing	Infectious disease risk Poor mental health Weather exposure	Affordable housing programs Improve sanitation in informal areas
Employment and working conditions	Low-wage, informal, high-risk jobs	Occupational injuries Lack of insurance Financial insecurity	Enforce safety laws Ensure labor rights and legal aid
Social integration and support	Social isolation and discrimination Lack of support network	Higher anxiety and depression rates Limited access to social services	Migrant support centers and inclusion programs Cultural exchange and community events
Legal and administrative barriers	Complex legal procedures Difficulty obtaining permits	Stress, limited access to care and protections Administrative exclusion	Streamline legal/documentation processes Provide targeted legal aid
Education access for children	Barriers to school enrollment Language gaps and discrimination	Lower achievement Social exclusion Long-term health impacts	Inclusive schooling and language support Anti-discrimination training for staff/students
Mental health and well-being	Trauma from displacement Loss of home Uncertainty	Higher PTSD, depression, and anxiety rates	Trauma-informed mental health services Culturally competent professionals
Public perception and policy	Negative stereotypes Political resistance Limited resource allocation	Reduced service access Higher stress Discrimination	Anti-stigma awareness campaigns Inclusive urban policies for equal access

## Leveraging urban resources to promote health equity and policy implications

Creating equitable urban environments that foster healthy longevity requires coordinated efforts to address both traditional and emerging health risk factors.

### Diet and nutrition

Addressing dietary disparities requires systemic interventions that enhance food affordability and access to nutritious options. Policies should support the development of urban food markets, subsidies for fresh produce, and community-driven food initiatives that empower local residents. Investments in urban agriculture, mobile grocery units, and healthy corner store programs can bridge gaps in food deserts. Fiscal measures, such as taxes on sugary drinks and incentives for healthy meal programs, can further encourage healthier diets, particularly in low-income areas. Comprehensive food labeling and restrictions on advertising unhealthy foods in public spaces and near schools are critical in shaping healthier choices.

### Physical activity

Inclusive urban design is essential to promote regular physical activity (everyday refers to only 8%) across all socioeconomic groups. Investments in pedestrian pathways, bike lanes, public parks, and recreational facilities should prioritize low-income neighborhoods that historically lack safe and accessible spaces. Enhancing public transit systems and fostering mixed-use developments can support walkability and active commuting. Policies must also address occupational physical activity, encouraging workplace initiatives that reduce sedentary behavior and offer fitness opportunities. While guidelines and policies typically focus on increasing leisure-time physical activity, this advice tends to be disproportionally adopted by individuals in higher socioeconomic groups [113]. An inclusive public health approach should also promote healthy occupational physical activity. This can be achieved by encouraging sit-to-stand transitions for sedentary workers and safeguarding the health of those engaged in strenuous tasks or prolonged physical activity at low metabolic intensity such as cleaning [114].

### Tobacco control

Comprehensive tobacco control must extend beyond individual behavior change and address systemic drivers, such as the density of tobacco outlets in disadvantaged areas. Policies should include stricter regulation of tobacco sales, higher taxes, and enhanced enforcement of smoke-free public spaces (novel public policies). A meta-analysis on novel public policies demonstrated effectiveness in reducing the prevalence of daily smoking [115], with an increasing support by the public over time [116]. Community-driven interventions that address economic stress, provide smoking cessation programs, and promote positive cultural norms around non-smoking can further reduce tobacco use. Educational initiatives should highlight the impact of secondhand smoke, particularly in multi-unit housing.

### Sleep health

Improving sleep health in urban areas requires multifaceted interventions. Expanding affordable housing programs to reduce overcrowding, improving neighborhood safety, and enforcing noise reduction policies are essential. School-based education on sleep hygiene and mental health services can mitigate the effects of academic and social stressors. Workplace policies that limit shift work and promote flexible schedules can help counteract sleep disruption, particularly for low-income workers. Public infrastructure improvements, such as better street lighting and green buffers to mitigate noise, can also support healthier sleep patterns.

### Healthcare access

Reducing urban health inequities necessitates addressing geographical, financial, and cultural barriers to healthcare. Despite shorter distances to facilities in cities compared to rural areas, travel times can still be lengthy due to traffic congestion and poorly planned transit networks. A key priority should be the further development of digital healthcare tools, including online consultations, e-prescriptions, e-registration systems, and AI-based solutions that can enhance service efficiency, accessibility, and personalized care [117]. Policies should focus on expanding primary care networks, implementing telemedicine solutions, and deploying mobile health clinics to underserved areas [118]. Big data represent an opportunity to identify underserved areas and expand primary care services by strengthening networks of local clinics, pharmacies, and general practitioners [119]. Coordination among primary care providers, specialist services, and public health initiatives is essential to ensure continuity of care and improve health outcomes. Integrated care models that foster collaboration between general practitioners, hospitals, and social services can address the complex health and social needs of urban populations. Strengthening communication channels, using shared electronic health records, and implementing case management approaches can further enhance care coordination and reduce health disparities [120]. Universal health insurance and reduced out-of-pocket costs can prevent medical impoverishment. Culturally competent healthcare providers and community health workers can improve outreach to migrant and minority populations. Gender-sensitive care, including access to same-gender healthcare providers, can enhance service utilization among women in conservative settings.

### Environmental pollution

Reducing environmental health risks demands coordinated action across sectors. Policies should prioritize air quality improvements by reducing vehicle emissions, expanding green spaces, and enhancing public transit options. Urban freight emissions can be minimized through sustainable logistics solutions, including local parcel pick-up points to encourage active last-mile transport. The “polluter pays” principle, as seen in tiered road pricing systems can be applied to curb industrial and vehicular pollution. Public transport systems should be inclusive and address diverse mobility needs. Most are designed for straightforward, peak-hour commutes, often overlooking the multi-stop trips common among those with caregiving role and women. Fare structures and routes should be adapted to better support frequent, short journeys, making public transit more accessible and equitable [121]. Water quality and waste management initiatives must address the needs of informal settlements to prevent waterborne diseases.

### Climate resilience and adaptation

Mitigating the health impacts of climate change requires both preventive and adaptive measures. Investments in renewable energy, heat-resistant infrastructure, and smart urban design can reduce urban heat island effects. Disaster preparedness plans, early warning systems, and cooling shelters should prioritize vulnerable populations. Community-based education programs can enhance climate resilience by raising awareness about the health risks associated with extreme weather events.

### Mental health services

Expanding mental health services requires structural and community-level interventions. Integrating mental health care into primary healthcare through routine screenings and training for general practitioners can improve early detection and treatment. Subsidized mental health services and peer support programs can reduce stigma and improve access for low-income populations. Community-based centers offering counseling, support groups, and home-based care can enhance social support and autonomy for individuals with mental health conditions. Suicide prevention programs should include local facilitators, such as teachers and community leaders, to foster early intervention and reduce isolation. Vulnerable groups, including migrants and those in poverty, should receive coordinated mental, physical, and social care to address their complex needs. Addressing social determinants such as housing insecurity and unemployment is also essential for improving mental well-being.

### Housing and basic services

Housing policies must address affordability, safety, and quality standards. Investments in affordable housing, energy-efficient upgrades, and community sanitation facilities are crucial. Access to clean water, functional sanitation, and inclusive public restrooms, particularly in LMICs, remains a top priority. Addressing energy insufficiency through subsidized energy-saving installations can reduce health risks linked to extreme temperatures.

#### Age-friendly and inclusive cities

Urban environments should be designed to support aging in place and intergenerational living. Policies should focus on creating accessible public spaces, barrier-free transportation, and housing that accommodates older adults and individuals with disabilities. Programs combining physical activity, cognitive training, and social engagement can promote healthy aging and delay cognitive decline. Public spaces such as libraries, parks, and community centers can foster social connections, reducing isolation among older adults and promoting mental well-being.

#### Governance and cross-sector collaboration

Achieving urban health equity requires participatory governance that involves local communities in policymaking. The"Health in All Policies"approach ensures that health considerations are integrated across sectors, such as housing, education, and transportation. Health impact assessments can guide decision-making by evaluating the health implications of policies and projects [122]. Multi-sectoral partnerships, joint budgeting, and data-driven monitoring can strengthen accountability and enhance policy effectiveness. Finally, an innovation ecosystem for urban health can be cultivated by creating dedicated spaces for experimentation and employing tactical urbanism, a strategy that develops short-term, low-cost, and scalable pilot projects to test new ideas [123]. When these initiatives prove successful, they can serve as a strong foundation for advocating long-term policy changes.

## Conclusions

Urbanization presents both challenges and opportunities for global health, particularly in rapidly expanding urban areas where health disparities are pronounced. The interplay between social determinants, environmental risk factors, and healthcare access underscores the urgent need for integrated, equitable, and sustainable urban planning strategies. Addressing traditional and emerging health risks requires a comprehensive, multi-sectoral approach that fosters inclusive policies, strengthens healthcare systems, and promotes healthier lifestyles across diverse populations. By implementing evidence-based interventions and embracing participatory governance, cities can mitigate health inequities, improve environmental resilience, and enhance the well-being of their residents. Achieving healthier, more equitable urban environments will ultimately depend on bold commitments to intersectoral collaboration, community engagement, and continuous adaptation to the evolving needs of urban populations.

## Data Availability

No datasets were generated or analysed during the current study.

## References

[1] Global Report on Urban Health (2016) Equitable healthier cities for sustainable development. World Health Organization, Geneva

[2] Compendium of WHO and other UN guidance in health and environment, 2024 update. World Health Organization, Geneva (2024)

[3] Alvarez-Galvez J, Ortega-Martin E, Carretero-Bravo J et al (2023) Social determinants of multimorbidity patterns: a systematic review. Front Public Health 11:1081518. 10.3389/fpubh.2023.1081518 37050950 10.3389/fpubh.2023.1081518 PMC10084932

[4] Rao M, Prasad S, Adshead F et al (2007) The built environment and health. Lancet 370:1111–1113. 10.1016/S0140-6736(07)61260-4 17868821 10.1016/S0140-6736(07)61260-4

[5] Seyedrezaei M, Becerik-Gerber B, Awada M et al (2023) Equity in the built environment: a systematic review. Build Environ. 10.1016/j.buildenv.2023.110827

[6] Suzman R, Beard JR, Boerma T et al (2015) Health in an ageing world–what do we know? Lancet 385:484–486. 10.1016/S0140-6736(14)61597-X 25468156 10.1016/S0140-6736(14)61597-X

[7] Kowal P, Kahn K, Ng N et al (2010) Ageing and adult health status in eight lower-income countries: the INDEPTH WHO-SAGE collaboration. Glob Health Action. 10.3402/gha.v3i0.5302 20959878 10.3402/gha.v3i0.5302 PMC2957285

[8] Garmany A, Terzic A (2024) Global healthspan-lifespan gaps among 183 world health organization member states. JAMA Netw Open. 10.1001/jamanetworkopen.2024.50241 39661386 10.1001/jamanetworkopen.2024.50241 PMC11635540

[9] Healthy life years statistics. Eurostat. Eurostat. 2024. https://ec.europa.eu/eurostat/statistics-explained/index.php?title=Healthy_life_years_statistics. Accessed 10 Jan 2025

[10] Chowdhury SR, Chandra Das D, Sunna TC et al (2023) Global and regional prevalence of multimorbidity in the adult population in community settings: a systematic review and meta-analysis. EClinicalMedicine 57:101860. 10.1016/j.eclinm.2023.101860 36864977 10.1016/j.eclinm.2023.101860 PMC9971315

[11] Strasser T (1978) Reflections on cardiovascular diseases. Interdisc Sci Rev 3:225–230. 10.1179/030801878791925921

[12] Hasbani NR, Ligthart S, Brown MR et al (2022) American Heart Association’s life’s simple 7: lifestyle recommendations, polygenic risk, and lifetime risk of coronary heart disease. Circulation 145:808–818. 10.1161/CIRCULATIONAHA.121.053730 35094551 10.1161/CIRCULATIONAHA.121.053730 PMC8912968

[13] Lloyd-Jones DM, Allen NB, Anderson CAM et al (2022) Life’s essential 8: updating and enhancing the American Heart Association’s construct of cardiovascular health: a presidential advisory from the American Heart Association. Circulation 146:e18–e43. 10.1161/CIR.0000000000001078 35766027 10.1161/CIR.0000000000001078 PMC10503546

[14] Kumar M, Orkaby A, Tighe C et al (2023) Life’s essential 8: optimizing health in older adults. JACC Adv. 10.1016/j.jacadv.2023.100560 37664644 10.1016/j.jacadv.2023.100560 PMC10470487

[15] Zhang Y, Sun M, Wang Y et al (2023) Association of cardiovascular health using Life’s Essential 8 with noncommunicable disease multimorbidity. Prev Med 174:107607. 10.1016/j.ypmed.2023.107607 37414227 10.1016/j.ypmed.2023.107607

[16] Zhou R, Chen HW, Li FR et al (2023) “Life’s Essential 8” cardiovascular health and dementia risk, cognition, and neuroimaging markers of brain health. J Am Med Dir Assoc 24:1791–1797. 10.1016/j.jamda.2023.05.023 37369360 10.1016/j.jamda.2023.05.023

[17] Banach M, Toth PP, Bielecka-Dabrowa A et al (2024) Primary and secondary cardiovascular prevention: recent advances. Kardiol Pol 82:1200–1210. 10.33963/v.phj.103997 39775556 10.33963/v.phj.103997

[18] van der Heide FCT, Valeri L, Dugravot A et al (2024) Role of cardiovascular health factors in mediating social inequalities in the incidence of dementia in the UK: two prospective, population-based cohort studies. EClinicalMedicine 70:102539. 10.1016/j.eclinm.2024.102539 38516105 10.1016/j.eclinm.2024.102539 PMC10955651

[19] Vilar-Compte M, Burrola-Mendez S, Lozano-Marrufo A et al (2021) Urban poverty and nutrition challenges associated with accessibility to a healthy diet: a global systematic literature review. Int J Equity Health 20:40. 10.1186/s12939-020-01330-0 33472636 10.1186/s12939-020-01330-0 PMC7816472

[20] MacIntyre UE, Kruger HS, Venter CS et al (2002) Dietary intakes of an African population in different stages of transition in the North West Province, South Africa: the THUSA study. Nutr Res 22:239–256. 10.1016/s0271-5317(01)00392-x

[21] Howard AG, Attard SM, Herring AH et al (2021) Socioeconomic gradients in the Westernization of diet in China over 20 years. SSM Popul Health 16:100943. 10.1016/j.ssmph.2021.100943 34703875 10.1016/j.ssmph.2021.100943 PMC8526760

[22] Denniss E, Lindberg R, Marchese LE et al (2024) #Fail: the quality and accuracy of nutrition-related information by influential Australian Instagram accounts. Int J Behav Nutr Phys Act 21:16. 10.1186/s12966-024-01565-y 38355567 10.1186/s12966-024-01565-y PMC10865719

[23] Kodur N, Yurista S, Province V et al (2023) Ketogenic diet in heart failure: fact or fiction? JACC Heart Fail 11:838–844. 10.1016/j.jchf.2023.05.009 37407158 10.1016/j.jchf.2023.05.009

[24] Kommu S, Berg RL (2024) Efficacy and safety of once-weekly subcutaneous semaglutide on weight loss in patients with overweight or obesity without diabetes mellitus—a systematic review and meta-analysis of randomized controlled trials. Obes Rev 25:e13792. 10.1111/obr.13792 38923272 10.1111/obr.13792

[25] Prado CM, Phillips SM, Gonzalez MC et al (2024) Muscle matters: the effects of medically induced weight loss on skeletal muscle. Lancet Diabetes Endocrinol 12:785–787. 10.1016/S2213-8587(24)00272-9 39265590 10.1016/S2213-8587(24)00272-9

[26] Chiappini S, Vickers-Smith R, Harris D et al (2023) Is There a risk for semaglutide misuse? Focus on the food and drug administration’s FDA adverse events reporting system (FAERS) pharmacovigilance dataset. Pharmaceuticals (Basel) 16:25. 10.3390/ph16070994 10.3390/ph16070994 PMC10384093 37513906

[27] Weissman YL, Calvarysky B, Shochat T et al (2024) Disparities in sodium-glucose cotransporter 2 (SGLT2) inhibitor prescription and dispensing in the Israeli population—a retrospective cohort study. Diabetes Care 47:692–697. 10.2337/dc23-1652 38377492 10.2337/dc23-1652

[28] Armstrong S, Wong CA, Perrin E et al (2018) Association of physical activity with income, race/ethnicity, and sex among adolescents and young adults in the United States: findings From the National Health and Nutrition Examination Survey, 2007–2016. JAMA Pediatr 172:732–740. 10.1001/jamapediatrics.2018.1273 29889945 10.1001/jamapediatrics.2018.1273 PMC6142913

[29] Sallis JF, Cerin E, Conway TL et al (2016) Physical activity in relation to urban environments in 14 cities worldwide: a cross-sectional study. Lancet 387:2207–2217. 10.1016/S0140-6736(15)01284-2 27045735 10.1016/S0140-6736(15)01284-2 PMC10833440

[30] Brownson RC, Baker EA, Housemann RA et al (2001) Environmental and policy determinants of physical activity in the United States. Am J Public Health 91:1995–2003. 10.2105/ajph.91.12.1995 11726382 10.2105/ajph.91.12.1995 PMC1446921

[31] Hinrichs T, Keskinen KE, Pavelka B et al (2019) Perception of parks and trails as mobility facilitators and transportation walking in older adults: a study using digital geographical maps. Aging Clin Exp Res 31:673–683. 10.1007/s40520-018-01115-0 30666515 10.1007/s40520-018-01115-0

[32] Glasser AM, Onnen N, Craigmile PF et al (2022) Associations between disparities in tobacco retailer density and disparities in tobacco use. Prev Med 154:106910. 10.1016/j.ypmed.2021.106910 34921833 10.1016/j.ypmed.2021.106910 PMC8750533

[33] Farley SM, Maroko AR, Suglia SF et al (2019) The influence of tobacco retailer density and poverty on tobacco use in a densely populated urban environment. Public Health Rep 134:164–171. 10.1177/0033354918824330 30763150 10.1177/0033354918824330 PMC6410483

[34] Patterson F, Seravalli L, Hanlon A et al (2012) Neighborhood safety as a correlate of tobacco use in a sample of urban, pregnant women. Addict Behav 37:1132–1137. 10.1016/j.addbeh.2012.05.011 22688344 10.1016/j.addbeh.2012.05.011 PMC3429339

[35] Widome R, Joseph AM, Hammett P et al (2015) Associations between smoking behaviors and financial stress among low-income smokers. Prev Med Rep 2:911–915. 10.1016/j.pmedr.2015.10.011 26844167 10.1016/j.pmedr.2015.10.011 PMC4721304

[36] Garritsen HH, Khan F, Rozema AD et al (2024) Associations of smoke-free policies in multi-unit housing with smoking behavior and second-hand smoke exposure: a systematic review. Addiction. 10.1111/add.16724 39639831 10.1111/add.16724 PMC11907329

[37] Awad K, Mohammed M, Martin SS et al (2023) Association between electronic nicotine delivery systems use and risk of stroke: a meta-analysis of 1,024,401 participants. Arch Med Sci 19:1538–1540. 10.5114/aoms/171473 37732043 10.5114/aoms/171473 PMC10507757

[38] Logue JM, Sleiman M, Montesinos VN et al (2017) Emissions from electronic cigarettes: assessing vapers’ intake of toxic compounds, secondhand exposures, and the associated health impacts. Environ Sci Technol 51:9271–9279. 10.1021/acs.est.7b00710 28766331 10.1021/acs.est.7b00710

[39] Barnes C, McCrabb S, Bialek C et al (2024) Factors associated with child and adolescent electronic nicotine and non-nicotine delivery systems use: a scoping review. Prev Med 181:107895. 10.1016/j.ypmed.2024.107895 38354861 10.1016/j.ypmed.2024.107895

[40] Grandner MA, Williams NJ, Knutson KL et al (2016) Sleep disparity, race/ethnicity, and socioeconomic position. Sleep Med 18:7–18. 10.1016/j.sleep.2015.01.020 26431755 10.1016/j.sleep.2015.01.020 PMC4631795

[41] Hunter JC, Hayden KM (2018) The association of sleep with neighborhood physical and social environment. Public Health 162:126–134. 10.1016/j.puhe.2018.05.003 30036811 10.1016/j.puhe.2018.05.003 PMC6269089

[42] Su TP, Huang SR, Chou P (2004) Prevalence and risk factors of insomnia in community-dwelling Chinese elderly: a Taiwanese urban area survey. Aust N Z J Psychiatry 38:706–713. 10.1080/j.1440-1614.2004.01444.x 15324335 10.1080/j.1440-1614.2004.01444.x

[43] Tang J, Liao Y, Kelly BC et al (2017) Gender and regional differences in sleep quality and insomnia: a general population-based study in Hunan Province of China. Sci Rep 7:43690. 10.1038/srep43690 28262807 10.1038/srep43690 PMC5337959

[44] MacKillop J, Agabio R, Feldstein Ewing SW et al (2022) Hazardous drinking and alcohol use disorders. Nat Rev Dis Primers 8:80. 10.1038/s41572-022-00406-1 36550121 10.1038/s41572-022-00406-1 PMC10284465

[45] GBD 2017 Risk Factor Collaborators Global, regional, and national comparative risk assessment of 84 behavioural, environmental and occupational, and metabolic risks or clusters of risks for 195 countries and territories, 1990–2017: a systematic analysis for the Global Burden of Disease Study 2017. Lancet 392(10159):1923–1994 (2018). 10.1016/S0140-6736(18)32225-6 10.1016/S0140-6736(18)32225-6 PMC6227755 30496105

[46] Slutske WS, Deutsch AR, Piasecki TM (2016) Neighborhood contextual factors, alcohol use, and alcohol problems in the united states: evidence from a nationally representative study of young adults. Alcohol Clin Exp Res 40:1010–1019. 10.1111/acer.13033 26996826 10.1111/acer.13033 PMC4844782

[47] Sundarakumar JS, Stezin A, Menesgere AL et al (2022) Rural-urban and gender differences in metabolic syndrome in the aging population from southern India: two parallel, prospective cohort studies. EClinicalMedicine 47:101395. 10.1016/j.eclinm.2022.101395 35497067 10.1016/j.eclinm.2022.101395 PMC9044001

[48] Crouch E, Abshire DA, Wirth MD et al (2023) Rural-urban differences in overweight and obesity, physical activity, and food security among children and adolescents. Prev Chronic Dis 20:E92. 10.5888/pcd20.230136 37857462 10.5888/pcd20.230136 PMC10599326

[49] Macicame I, Prista A, Parhofer KG et al (2021) Social determinants and behaviors associated with overweight and obesity among youth and adults in a peri-urban area of Maputo City, Mozambique. J Glob Health 11:04021. 10.7189/jogh.11.04021 33868672 10.7189/jogh.11.04021 PMC8038757

[50] Ajayi IO, Adebamowo C, Adami HO et al (2016) Urban-rural and geographic differences in overweight and obesity in four sub-Saharan African adult populations: a multi-country cross-sectional study. BMC Public Health 16:1126. 10.1186/s12889-016-3789-z 27793143 10.1186/s12889-016-3789-z PMC5084330

[51] Talukder A, Sara SS, Khan ZI et al (2024) Prevalence and determinants of hypertension in South-Asian Urban Communities: findings from Demographic and Health Surveys (DHS) data of South Asian countries. J Hum Hypertens 38:257–266. 10.1038/s41371-023-00879-x 38049636 10.1038/s41371-023-00879-x

[52] Walker RJ, Garacci E, Palatnik A et al (2020) The longitudinal influence of social determinants of health on glycemic control in elderly adults with diabetes. Diabetes Care 43:759–766. 10.2337/dc19-1586 32029639 10.2337/dc19-1586 PMC7085811

[53] Ghazwani M, Mahmood SE, Gosadi IM et al (2023) Prevalence of dyslipidemia and its determinants among the adult population of the Jazan Region. Int J Gen Med 16:4215–4226. 10.2147/IJGM.S429462 37745134 10.2147/IJGM.S429462 PMC10516128

[54] Adhikari P, Sriyuktasuth A, Phligbua W (2023) Social determinants of health and glycemic control in persons with type 2 diabetes mellitus attending a tertiary hospital in Nepal: a cross-sectional study. Belitung Nurs J 9:489–497 37901380 10.33546/bnj.2753 PMC10600700

[55] Silva-Tinoco R, Cuatecontzi-Xochitiotzi T, De la Torre-Saldana V et al (2020) Influence of social determinants, diabetes knowledge, health behaviors, and glycemic control in type 2 diabetes: an analysis from real-world evidence. BMC Endocr Disord 20:130. 10.1186/s12902-020-00604-6 32843004 10.1186/s12902-020-00604-6 PMC7449009

[56] Esteghamati A, Ismail-Beigi F, Khaloo P et al (2020) Determinants of glycemic control: phase 2 analysis from nationwide diabetes report of National Program for Prevention and Control of Diabetes (NPPCD-2018). Prim Care Diabetes 14:222–231. 10.1016/j.pcd.2019.07.002 31402326 10.1016/j.pcd.2019.07.002

[57] Liu MY, Li N, Li WA et al (2017) Association between psychosocial stress and hypertension: a systematic review and meta-analysis. Neurol Res 39:573–580. 10.1080/01616412.2017.1317904 28415916 10.1080/01616412.2017.1317904

[58] Mankar A, Kawalkar U, Jadhao N et al (2024) Exploring the interplay of socioeconomic and behavioral factors: unraveling gender disparities in glycemic control among adult type 2 diabetic patients in outpatient care. Cureus 16:e56505. 10.7759/cureus.56505 38646396 10.7759/cureus.56505 PMC11026148

[59] Subasinghe AK, Arabshahi S, Busingye D et al (2016) Association between salt and hypertension in rural and urban populations of low to middle income countries: a systematic review and meta-analysis of population based studies. Asia Pac J Clin Nutr 25:402–413. 10.6133/apjcn.2016.25.2.25 27222425 10.6133/apjcn.2016.25.2.25

[60] Opoku S, Gan Y, Fu W et al (2019) Prevalence and risk factors for dyslipidemia among adults in rural and urban China: findings from the China National Stroke Screening and prevention project (CNSSPP). BMC Public Health 19:1500. 10.1186/s12889-019-7827-5 31711454 10.1186/s12889-019-7827-5 PMC6849283

[61] Basit A, Sabir S, Riaz M et al (2020) members N: NDSP 05: Prevalence and pattern of dyslipidemia in urban and rural areas of Pakistan; a sub analysis from second National Diabetes Survey of Pakistan (NDSP) 2016–2017. J Diabetes Metab Disord 19:1215–1225. 10.1007/s40200-020-00631-z 33520835 10.1007/s40200-020-00631-z PMC7843689

[62] Lazo-Porras M, Bernabe-Ortiz A, Quispe R et al (2017) Urbanization, mainly rurality, but not altitude is associated with dyslipidemia profiles. J Clin Lipidol 11:1212-1222 e1214. 10.1016/j.jacl.2017.06.016 28780399 10.1016/j.jacl.2017.06.016 PMC5624786

[63] Shin J, Cho KH, Choi Y et al (2016) Combined effect of individual and neighborhood socioeconomic status on mortality in patients with newly diagnosed dyslipidemia: a nationwide Korean cohort study from 2002 to 2013. Nutr Metab Cardiovasc Dis 26:207–215. 10.1016/j.numecd.2015.12.007 26895648 10.1016/j.numecd.2015.12.007

[64] Studzinski K, Tomasik T, Windak A et al (2021) The differences in the prevalence of cardiovascular disease, its risk factors, and achievement of therapeutic goals among urban and rural primary care patients in poland: results from the LIPIDOGRAM 2015 study. J Clin Med. 10.3390/jcm10235656 34884357 10.3390/jcm10235656 PMC8658414

[65] Burzynska M, Jankowski P, Babicki M et al (2024) Prevalence of hyperlipoproteinemia(a) in individuals of European ancestry treated at outpatient cardiology clinics: results from a cross-sectional STAR-Lp(a) study. Pol Arch Intern Med. 10.20452/pamw.16860 39352310 10.20452/pamw.16860

[66] Sosnowska B, Lewek J, Adach W et al (2024) The prevalence, patients’ characteristics, and hyper-Lp(a)-emia risk factors in the Polish population. The first results from the PMMHRI-Lp(a) Registry. Prog Cardiovasc Dis 86:54–61. 10.1016/j.pcad.2024.08.004 39191356 10.1016/j.pcad.2024.08.004

[67] Montone RA, Camilli M, Calvieri C et al (2024) Exposome in ischaemic heart disease: beyond traditional risk factors. Eur Heart J 45:419–438. 10.1093/eurheartj/ehae001 38238478 10.1093/eurheartj/ehae001 PMC10849374

[68] Rajagopalan S, Al-Kindi SG, Brook RD (2018) Air Pollution and cardiovascular disease: JACC state-of-the-art review. J Am Coll Cardiol 72:2054–2070. 10.1016/j.jacc.2018.07.099 30336830 10.1016/j.jacc.2018.07.099

[69] Guan WJ, Zheng XY, Chung KF et al (2016) Impact of air pollution on the burden of chronic respiratory diseases in China: time for urgent action. Lancet 388:1939–1951. 10.1016/S0140-6736(16)31597-5 27751401 10.1016/S0140-6736(16)31597-5

[70] Ning P, Guo X, Qu Q et al (2023) Exploring the association between air pollution and Parkinson’s disease or Alzheimer’s disease: a Mendelian randomization study. Environ Sci Pollut Res Int 30:123939–123947. 10.1007/s11356-023-31047-w 37995032 10.1007/s11356-023-31047-w

[71] Fussell JC, Jauniaux E, Smith RB et al (2024) Ambient air pollution and adverse birth outcomes: a review of underlying mechanisms. BJOG 131:538–550. 10.1111/1471-0528.17727 38037459 10.1111/1471-0528.17727 PMC7615717

[72] Zhang T, Liu W, Yang T et al (2024) Association between ambient fine particular matter components and subsequent cognitive impairment in community-dwelling older people: a prospective cohort study from eastern China. Aging Clin Exp Res 36:150. 10.1007/s40520-024-02793-9 39060791 10.1007/s40520-024-02793-9 PMC11282123

[73] Stewart JA, Mitchell MA, Edgerton VS et al (2015) Environmental justice and health effects of urban air pollution. J Natl Med Assoc 107:50–58. 10.1016/S0027-9684(15)30009-2 27282527 10.1016/S0027-9684(15)30009-2

[74] Bennitt FB, Wozniak SS, Causey K et al (2021) Estimating disease burden attributable to household air pollution: new methods within the Global Burden of Disease Study. Lancet Global Health. 10.1016/s2214-109x(21)00126-1

[75] Pinho-Gomes AC, Roaf E, Fuller G et al (2023) Air pollution and climate change. Lancet Planet Health 7:e727–e728. 10.1016/S2542-5196(23)00189-4 37673539 10.1016/S2542-5196(23)00189-4

[76] Heaviside C, Macintyre H, Vardoulakis S (2017) The urban heat island: implications for health in a changing environment. Curr Environ Health Rep 4:296–305. 10.1007/s40572-017-0150-3 28695487 10.1007/s40572-017-0150-3

[77] Alho AM, Oliveira AP, Viegas S et al (2024) Effect of heatwaves on daily hospital admissions in Portugal, 2000–18: an observational study. Lancet Planet Health 8:e318–e326. 10.1016/S2542-5196(24)00046-9 38729671 10.1016/S2542-5196(24)00046-9

[78] Buguet A, Radomski MW, Reis J et al (2023) Heatwaves and human sleep: stress response versus adaptation. J Neurol Sci 454:120862. 10.1016/j.jns.2023.120862 37922826 10.1016/j.jns.2023.120862

[79] Fu X, Wang L, Yuan L et al (2023) Long-term exposure to traffic noise and risk of incident cardiovascular diseases: a systematic review and dose-response meta-analysis. J Urban Health 100:788–801. 10.1007/s11524-023-00769-0 37580544 10.1007/s11524-023-00769-0 PMC10447855

[80] Cai Y, Ramakrishnan R, Rahimi K (2021) Long-term exposure to traffic noise and mortality: a systematic review and meta-analysis of epidemiological evidence between 2000 and 2020. Environ Pollut 269:116222. 10.1016/j.envpol.2020.116222 33307398 10.1016/j.envpol.2020.116222

[81] Lin L, Yang H, Xu X (2022) Effects of water pollution on human health and disease heterogeneity: a review. Front Environ Sci. 10.3389/fenvs.2022.880246

[82] Oli N, Vaidya A, Thapa G (2013) Behavioural risk factors of noncommunicable diseases among nepalese urban poor: a descriptive study from a slum area of kathmandu. Epidemiol Res Int 2013:1–13. 10.1155/2013/329156

[83] Lima R, Batalha MA, Ribeiro CCC et al (2023) Modifiable behavioral risk factors for NCDs and sleep in Brazilian adolescents. Rev Saude Publ 57:60. 10.11606/s1518-8787.2023057004957 10.11606/s1518-8787.2023057004957 PMC10519685 37878846

[84] Barbosa JMA, Ribeiro CCC, Batista RFL et al (2022) Behavioral risk factors for noncommunicable diseases associated with depression and suicide risk in adolescence. Cad Saude Publica 38:e00055621. 10.1590/0102-311X00055621 35293517 10.1590/0102-311X00055621

[85] Merrill AK, Sobolewski M, Susiarjo M (2023) Exposure to endocrine disrupting chemicals impacts immunological and metabolic status of women during pregnancy. Mol Cell Endocrinol 577:112031. 10.1016/j.mce.2023.112031 37506868 10.1016/j.mce.2023.112031 PMC10592265

[86] Song Y, Chou EL, Baecker A et al (2016) Endocrine-disrupting chemicals, risk of type 2 diabetes, and diabetes-related metabolic traits: a systematic review and meta-analysis. J Diabetes 8:516–532. 10.1111/1753-0407.12325 26119400 10.1111/1753-0407.12325

[87] Predieri B, Iughetti L, Bernasconi S et al (2022) Endocrine disrupting chemicals’ effects in children: what we know and what we need to learn? Int J Mol Sci. 10.3390/ijms231911899 36233201 10.3390/ijms231911899 PMC9570268

[88] Parvez SM, Jahan F, Brune MN et al (2021) Health consequences of exposure to e-waste: an updated systematic review. Lancet Planet Health 5:e905–e920. 10.1016/S2542-5196(21)00263-1 34895498 10.1016/S2542-5196(21)00263-1 PMC8674120

[89] Aboukorin SAA, Han H, Mahran MGN (2021) Role of urban planning characteristics in forming pandemic resilient cities—case study of Covid-19 impacts on European cities within England, Germany and Italy. Cities 118:103324. 10.1016/j.cities.2021.103324 34539022 10.1016/j.cities.2021.103324 PMC8435089

[90] Hassell JM, Begon M, Ward MJ et al (2017) Urbanization and disease emergence: dynamics at the wildlife–livestock–human interface. Trends Ecol Evol 32:55–67. 10.1016/j.tree.2016.09.012 28029378 10.1016/j.tree.2016.09.012 PMC5214842

[91] Anzalone AJ, Jackson LE, Singh N et al (2025) Long-term mortality following SARS-CoV-2 infection in rural versus urban dwellers with autoimmune or inflammatory rheumatic disease: a retrospective cohort analysis from the national COVID cohort collaborative. Arthritis Care Res (Hoboken) 77:143–155. 10.1002/acr.25421 39158165 10.1002/acr.25421 PMC11693476

[92] Sunkersing D, Goodfellow H, Mu Y et al (2024) Long COVID symptoms and demographic associations: a retrospective case series study using healthcare application data. JRSM Open 15:20542704241274292. 10.1177/20542704241274292 39228407 10.1177/20542704241274292 PMC11367609

[93] Weilnhammer V, Schmid J, Mittermeier I et al (2021) Extreme weather events in europe and their health consequences—a systematic review. Int J Hyg Environ Health 233:113688. 10.1016/j.ijheh.2021.113688 33530011 10.1016/j.ijheh.2021.113688

[94] Chaudhry D (2024) Climate change and health of the urban poor: The role of environmental justice. J Clim Change Health. 10.1016/j.joclim.2023.100277 10.1016/j.joclim.2023.100277 PMC12851302 41646043

[95] Xie Y, Guo Q, Meng Y (2021) The health service use of aged rural-to-urban migrant workers in different types of cities in China. BMC Health Serv Res 21:606. 10.1186/s12913-021-06638-3 34182984 10.1186/s12913-021-06638-3 PMC8237433

[96] Thompson B, Molina Y, Viswanath K et al (2016) Strategies to empower communities to reduce health disparities. Health Aff (Millwood) 35:1424–1428. 10.1377/hlthaff.2015.1364 27503967 10.1377/hlthaff.2015.1364 PMC5554943

[97] Cacciatore S, Spadafora L, Andaloro S et al (2024) Healthy aging and cardiovascular health in Kyrgyzstan: current status and emerging challenges. Heart Vessels Transp. 10.24969/hvt.2024.462

[98] Khan HTA, Addo KM, Findlay H (2024) Public health challenges and responses to the growing ageing populations. Public Health Chall. 10.1002/puh2.213 10.1002/puh2.213 PMC12039680 40496520

[99] Preda M, Vijulie I, Lequeux-Dincă A-I et al (2022) How do the new residential areas in Bucharest satisfy population demands, and where do they fall short? Land. 10.3390/land11060855

[100] Baldwin C, Matthews T, Byrne J (2020) Planning for older people in a rapidly warming and ageing world: the role of urban greening. Urban Policy Res 38:199–212. 10.1080/08111146.2020.1780424

[101] Fang EF, Xie C, Schenkel JA et al (2020) A research agenda for ageing in China in the 21st century (2nd edition): focusing on basic and translational research, long-term care, policy and social networks. Ageing Res Rev 64:101174. 10.1016/j.arr.2020.101174 32971255 10.1016/j.arr.2020.101174 PMC7505078

[102] Xie B, Charness N, Fingerman K et al (2020) When going digital becomes a necessity: ensuring older adults’ needs for information, services, and social inclusion during COVID-19. J Aging Soc Policy 32:460–470. 10.1080/08959420.2020.1771237 32507061 10.1080/08959420.2020.1771237 PMC8855980

[103] Rahaman MA, Kalam A, Al-Mamun M (2023) Unplanned urbanization and health risks of Dhaka City in Bangladesh: uncovering the associations between urban environment and public health. Front Public Health 11:1269362. 10.3389/fpubh.2023.1269362 37927876 10.3389/fpubh.2023.1269362 PMC10620720

[104] Mathew B, Nambiar D (2020) Understanding the experiences of health care-seeking migrants in Delhi: trajectories and challenges. Qual Health Res 30:1710–1722. 10.1177/1049732320921374 32449456 10.1177/1049732320921374

[105] Caidi N, Du JT, Li L et al (2020) Immigrating after 60: Information experiences of older Chinese migrants to Australia and Canada. Inf Process Manag. 10.1016/j.ipm.2019.102111

[106] Jang SY, Oksuzyan A, Myrskyla M et al (2023) Healthy immigrants, unhealthy ageing? Analysis of health decline among older migrants and natives across European countries. SSM Popul Health 23:101478. 10.1016/j.ssmph.2023.101478 37635989 10.1016/j.ssmph.2023.101478 PMC10448331

[107] Cova I, Del Tedesco F, Maggiore L et al (2020) Cognitive disorders in migrants: retrospective analysis in a Center for Cognitive Disorders and Dementia in Milan. Aging Clin Exp Res 32:535–538. 10.1007/s40520-019-01224-4 31131428 10.1007/s40520-019-01224-4

[108] Nieboer AP, Cramm JM (2024) Growing old outside of one’s home country: Well-being needs for aging in place among Turkish people in the Netherlands. Cities. 10.1016/j.cities.2024.105065

[109] Di Nolfi A, Giusti A, Canevelli M et al (2024) Dementia among migrants in Italy: a qualitative study of the ImmiDem project. Ann Ist Super Sanita 60:264–273. 10.4415/ANN_24_04_05 39699980 10.4415/ANN_24_04_05

[110] Schoenmakers B, Robben T (2021) Barriers in screening for dementia in elderly migrants in primary care and the use of the Rowland Universal Dementia Assessment Scale. A mixed cross-sectional and qualitative study. Eur J Gen Pract 27:45–50. 10.1080/13814788.2021.1913116 33928835 10.1080/13814788.2021.1913116 PMC8816395

[111] Biswas S, Sun W, Stanyon W et al (2022) Exploring the factors that influence equitable access to and social participation in dementia care programs by foreign-born population living in Toronto and Durham region. Aging Clin Exp Res 34:2231–2235. 10.1007/s40520-022-02136-6 35534763 10.1007/s40520-022-02136-6

[112] Berdai Chaouni S, Smetcoren AS, De Donder L (2020) Caring for migrant older Moroccans with dementia in Belgium as a complex and dynamic transnational network of informal and professional care: a qualitative study. Int J Nurs Stud 101:103413. 10.1016/j.ijnurstu.2019.103413 31678839 10.1016/j.ijnurstu.2019.103413

[113] Straker L, Holtermann A, Lee IM et al (2020) Privileging the privileged: the public health focus on leisure time physical activity has contributed to widening socioeconomic inequalities in health. Br J Sports Med. 10.1136/bjsports-2020-103356 33004406 10.1136/bjsports-2020-103356

[114] Straker L, Mathiassen SE, Holtermann A (2018) The “Goldilocks Principle”: designing physical activity at work to be “just right” for promoting health. Br J Sports Med 52:818–819. 10.1136/bjsports-2017-097765 28663212 10.1136/bjsports-2017-097765 PMC6029635

[115] Darzi A, Keown OP, Chapman S (2015) Is a smoking ban in UK parks and outdoor spaces a good idea? BMJ 350:h958. 10.1136/bmj.h958 25715970 10.1136/bmj.h958

[116] Boderie NW, Sheikh A, Lo E et al (2023) Public support for smoke-free policies in outdoor areas and (semi-)private places: a systematic review and meta-analysis. EClinicalMedicine 59:101982. 10.1016/j.eclinm.2023.101982 37256097 10.1016/j.eclinm.2023.101982 PMC10225670

[117] Yeung AWK, Torkamani A, Butte AJ et al (2023) The promise of digital healthcare technologies. Front Public Health 11:1196596. 10.3389/fpubh.2023.1196596 37822534 10.3389/fpubh.2023.1196596 PMC10562722

[118] Jamir L, Nongkynrih B, Gupta SK (2013) Mobile health clinics: meeting health needs of the urban underserved. Indian J Community Med 38:132–134. 10.4103/0970-0218.116347 24019596 10.4103/0970-0218.116347 PMC3760319

[119] Yang Y, Cho A, Nguyen Q et al (2023) Association of neighborhood racial and ethnic composition and historical redlining with built environment indicators derived from street view images in the US. JAMA Netw Open 6:e2251201. 10.1001/jamanetworkopen.2022.51201 36652250 10.1001/jamanetworkopen.2022.51201 PMC9856713

[120] The Lancet Regional Health—Europe The Italian health data system is broken. Lancet Reg Health Europe 2025. 10.1016/j.lanepe.2024.101206 10.1016/j.lanepe.2024.101206 PMC11980697 40206218

[121] Epker E. Cities are designed for men’s convenience—not for women’s health: forbes. https://www.forbes.com. Accessed 2 Jan 2025

[122] McDermott R, Douglas MJ, Haigh F et al (2024) A systematic review of whether Health Impact Assessment frameworks support best practice principles. Public Health 233:137–144. 10.1016/j.puhe.2024.05.008 38878738 10.1016/j.puhe.2024.05.008

[123] Silva P (2016) Tactical urbanism: towards an evolutionary cities’ approach? Environ Plann B Plann Des 43:1040–1051. 10.1177/0265813516657340

